# Supplementary material for: COZOID: contact zone identifier for visual analysis of protein-protein interactions
Source: BMC Bioinformatics. 2018 Apr 6;19:125. doi: 10.1186/s12859-018-2113-6 (PMC5889581; doi:10.1186/s12859-018-2113-6)
Supplement: Supplementary file 2 — Software build. Executable binary file of the software tool. (ZIP 118,784 kb) [file 12859_2018_2113_MOESM2_ESM.zip › cozoid/java/docs/beansbinding-1.2.1-doc.zip!/org/jdesktop/observablecollections/ObservableMapListener.html]

ObservableMapListener (Beans Binding)


---


|  |  |  |
| --- | --- | --- |
| **Overview** \| **Package** \| **Class** \| **Use** \| **Tree** \| **Deprecated** \| **Index** \| **Help** | |  |
| **PREV CLASS**   NEXT CLASS | **FRAMES**    **NO FRAMES**     **All Classes** |  |
| SUMMARY: NESTED \| FIELD \| CONSTR \| METHOD | DETAIL: FIELD \| CONSTR \| METHOD |  |


---


## org.jdesktop.observablecollections Interface ObservableMapListener

---

``` public interface ObservableMapListener ```

Notification types from an `ObservableMap`.

---

| **Method Summary** | |
| --- | --- |
| `void` | `mapKeyAdded(ObservableMap map, java.lang.Object key)`             Notification that a key has been added. |
| `void` | `mapKeyRemoved(ObservableMap map, java.lang.Object key, java.lang.Object value)`             Notification that a key has been removed |
| `void` | `mapKeyValueChanged(ObservableMap map, java.lang.Object key, java.lang.Object lastValue)`             Notification that the value of an existing key has changed. |

**Method Detail**

### mapKeyValueChanged

```
void mapKeyValueChanged(ObservableMap map,
                        java.lang.Object key,
                        java.lang.Object lastValue)
```

:   Notification that the value of an existing key has changed.

    :   **Parameters:**: `map` - the `ObservableMap` that changed: `key` - the key: `lastValue` - the previous value

---


### mapKeyAdded

```
void mapKeyAdded(ObservableMap map,
                 java.lang.Object key)
```

:   Notification that a key has been added.

    :   **Parameters:**: `map` - the `ObservableMap` that changed: `key` - the key

---


### mapKeyRemoved

```
void mapKeyRemoved(ObservableMap map,
                   java.lang.Object key,
                   java.lang.Object value)
```

:   Notification that a key has been removed

    :   **Parameters:**: `map` - the `ObservableMap` that changed: `key` - the key: `value` - value for key before key was removed


---


|  |  |  |
| --- | --- | --- |
| **Overview** \| **Package** \| **Class** \| **Use** \| **Tree** \| **Deprecated** \| **Index** \| **Help** | |  |
| **PREV CLASS**   NEXT CLASS | **FRAMES**    **NO FRAMES**     **All Classes** |  |
| SUMMARY: NESTED \| FIELD \| CONSTR \| METHOD | DETAIL: FIELD \| CONSTR \| METHOD |  |


---
